# End-Organ Damage in Hypertension: An Insight on a Differentiated Outpatient Consultation

**DOI:** 10.7759/cureus.45105

**Published:** 2023-09-12

**Authors:** Rui Flores, Joana Lopes, Sofia Caridade

**Affiliations:** 1 Cardiology, Hospital de Braga, Braga, PRT; 2 Internal Medicine, Hospital de Braga, Braga, PRT

**Keywords:** public health and safety, renal hypertension, obstructive sleep apnea, renovascular hypertension, hypertension

## Abstract

Objective: The objective of this study was to determine the prevalence of end-organ damage in hypertensive patients attending an outpatient consultation.

Materials and methods: Patients were selected from an outpatient consultation at a tertiary hospital care center. All patients who consulted between July 2022 and March 2023 were included. Data on demographic characteristics, blood pressure records, hypertension etiology, medication use, and the presence of target organ damage were collected.

Results: A total of 73 patients were included in the study, with 34 patients being male (46.6%) and 39 patients being female (53.4%). The mean age of the patients was 49.8 years. Among the cases of hypertension, 14 (19.2%) were classified as secondary arterial hypertension (AH). The most common cause of secondary AH was obstructive sleep apnea (OSA) (42.9%). Approximately 23.2% of patients had documented end-organ damage potentially related to hypertension, with kidney disease being the most frequent (n = 10, 13.7%). The most commonly prescribed pharmacological classes were angiotensin-converting enzyme inhibitors and angiotensin II receptor antagonists (n = 46, 63%).

Conclusion: Despite numerous studies and trials on arterial hypertension, it remains a significant contributor to morbidity and mortality, necessitating the continued awareness of its long-term implications.

## Introduction

Blood pressure is the most commonly measured hemodynamic parameter due to its easy accessibility, but it only represents a small fraction of the overall state of the systemic vasculature [1]. Arterial hypertension (AH) is a significant contributor to morbidity and mortality globally, particularly in developed countries [2]. In the Western world, AH is highly prevalent, affecting approximately 30% of the population, with its incidence increasing with age [3]. Despite extensive research, many mechanisms underlying arterial hypertension remain unclear [3-7].

Since hypertension is often asymptomatic, many patients, especially younger individuals, underestimate the importance of this condition in society. Therefore, our main objective is to determine the prevalence of end-organ damage in hypertensive patients attending outpatient consultations in order to raise awareness of this public health issue.

## Materials and methods

We conducted a retrospective study at a single-center tertiary hospital, where we selected patients from a specialized consultation for arterial hypertension conducted by an internal medicine physician. The hospital's criteria for referring patients to hypertension consultations include the presence of hypertension at a young age, suspected secondary causes of hypertension, or difficult-to-control hypertension. The evaluation of the secondary forms of hypertension was carried out based on clinical suspicion, particularly in young patients with evidence of end-organ damage, as well as in cases of refractory or resistant hypertension and progressive deterioration of blood pressure profile and/or renal function despite escalated antihypertensive treatment, or when indicated by objective examination.

All patients who sought consultation between July 2022 and March 2023 were included in the study. Patients with incomplete registration data or those who had passed away at the time of data collection were excluded. We collected information on demographic data, blood pressure records, the underlying cause of hypertension, medication usage, and the presence of target organ damage. Verbal informed consent was obtained through telephone communication.

Our objective was to characterize the population of patients with arterial hypertension in a specialized hospital consultation, including the assessment of the prevalence of secondary forms of the disease, the incidence of end-organ damage, and the most commonly prescribed pharmacological classes.

## Results

For a nine-month follow-up period, 73 patients were evaluated in a total of 103 consultations. No patients were excluded. Of the total of 73 patients, 34 were males (46.6%), and 39 (53.4%) were females. The mean age was 49.8 years with a median age of 45 years.

**Figure 1:**
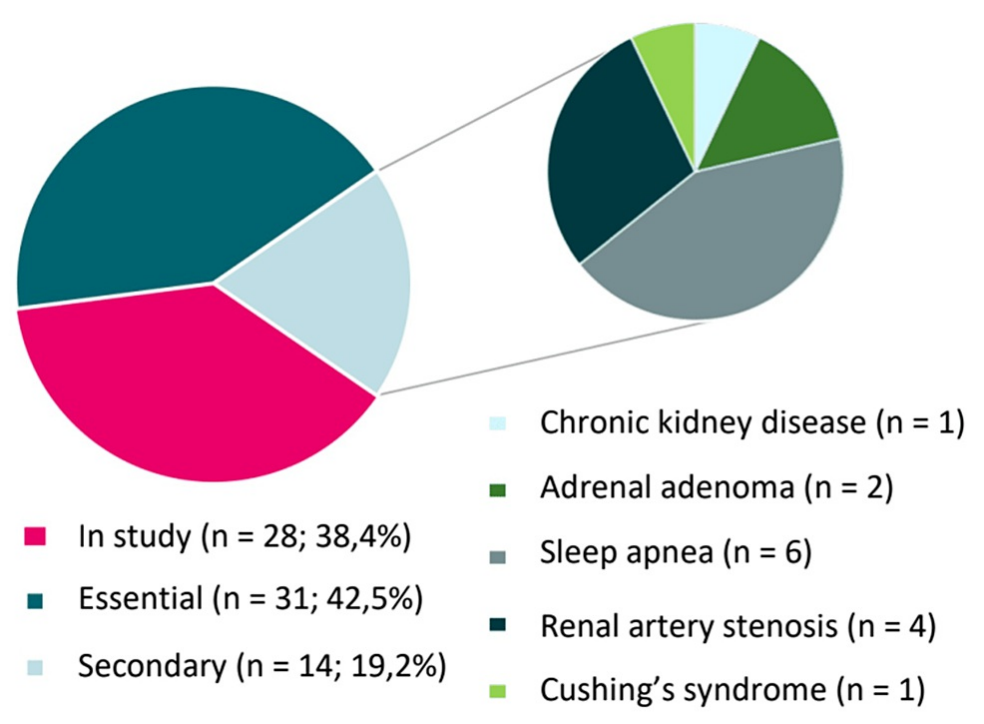
Distribution of the causes of AH in our sample. AH: arterial hypertension

Figure 1 showcases the distribution of the causes of AH.

We found 31 cases (42.5%) of essential and 14 (19.2%) of secondary AH. The remainder still had no established etiology by the time of data collection. In the group of secondary hypertension, six patients had obstructive sleep apnea (OSA) (42.9%), four had renal artery stenosis (28.6%), two had aldosterone-producing adrenal adenomas (14.3%), one had chronic kidney disease (7.1%), and one had Cushing's syndrome (7.1%).

**Figure 2:**
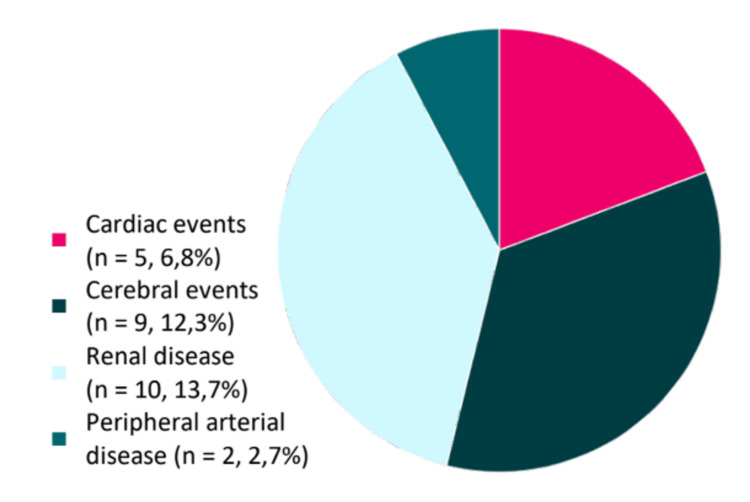
Distribution of macrovascular complications of AH. AH: arterial hypertension

Figure 2 shows the distribution of end-organ damage(s) in our patients.

A total of 23.2% (n = 17) had end-organ damage(s) potentially related to AH. Of these events, there was a higher proportion of chronic kidney disease (n = 10, 13.7%), followed by cerebrovascular events, namely, stroke (either ischemic or hemorrhagic, n = 9 {12.3%}), cardiac events (mainly coronary disease complications, n = 5 {6.8%}), and peripheral arterial disease (n = 2, 2.7%).

Table 1 shows the antihypertensive drugs used in our patients.

**Table 1:** Distribution of antihypertensive drugs used in our patients.

Antihypertensive drug class	n	%
Angiotensin-converting enzyme inhibitors or angiotensin II receptor antagonists	46	63
Calcium channel antagonists	43	58.9
Thiazide diuretics	36	49.3
Beta-blocker	20	27.2
Mineralocorticoid receptor antagonist	2	2.7
Central adrenergic agonist	2	2.7

The most used pharmacological class, in monotherapy or in combination, was angiotensin-converting enzyme inhibitors or angiotensin II receptor antagonists (n = 46, 63%). In descending order, the remaining classes were distributed as follows: calcium channel antagonists (n = 43, 58.9%), thiazide diuretics (n = 36, 49.3%), beta-blocker (n = 20, 27.2%), mineralocorticoid receptor antagonist (n = 2, 2.7%), and central adrenergic agonist (n = 2, 2.7%).

## Discussion

AH remains an important cause of morbidity and mortality, highlighting the need for research to discover new pharmacological classes [4]. The risk of target organ damage is directly proportional to uncontrolled blood pressure and increases over time [4,7-9].

Our sample exhibited a bimodal age distribution, reflecting two extremes of referral motives: young patients requiring the exclusion of potentially curable secondary causes of AH and older patients needing a wider range of therapies for blood pressure control. The strict control of blood pressure, particularly in younger age groups, is of paramount importance as it directly affects the risk of end-organ damage and the patient's quality of life. In our sample, a high percentage of patients showed end-organ damage, highlighting the significance of initiating tensional control at a young age to prevent AH complications.

Approximately 90% or more of AH cases have an unknown cause, categorized as idiopathic or primary AH [7]. The remaining cases correspond to secondary forms of AH [4,6,7]. In our sample, around 20% of patients were diagnosed with secondary AH, potentially underestimated due to ongoing investigations. The lower prevalence of secondary hypertension emphasizes the importance of maintaining specialized consultations for improved diagnostic and therapeutic management. The literature suggests that secondary AH may be directly or indirectly related to autonomic dysregulation and aldosterone overproduction [4,6,7]. These mechanisms appear intertwined with various secondary forms of AH, including metabolic syndrome and obesity [7]. In the case of obesity, animal models suggest a connection between the adipocyte production of angiotensin II and the vasculopathy of AH, raising questions about the potential role of neuraxial blockers in obese patients with AH [7]. Metabolic syndrome is also associated with an increased individual risk of endothelial dysfunction [10].

Models exploring the pathophysiology of AH are complex, involving the interaction of extrinsic and intrinsic factors, making it difficult to determine the relative importance of each factor [3,6-9]. Immune mechanisms appear correlated with micro- or macrovascular complications [9]. Prolonged hypertension leads to the systemic release of cytokines, including interleukin 17, interferon gamma, tumor necrosis factor-α, and interleukin 6, which contribute to renal fibrosis, alter sodium membrane transportation, and increase oxidative stress and arterial stiffness [9]. These cellular mechanisms are triggered by complement activation [3]. Therefore, our approach to hypertension, focusing on strict blood pressure control, goes beyond numbers and aims to prevent end-organ failure by reducing local and systemic inflammation triggers [3,9]. Conversely, several studies indicate the decreased secretion of anti-inflammatory molecules at the tissue level, exacerbating the pro-fibrotic and inflammatory components inherent in arterial hypertension [5].

The central role of angiotensin II and aldosterone in blood pressure regulation and as mediators of target organ damage makes the renin-angiotensin-aldosterone system a particularly promising therapeutic target [4,6,7]. In line with the literature, a majority of our patients were using agents that block this system, reflecting their importance in achieving effective blood pressure control and protecting against end-organ damage [11]. The efficacy of these agents has led to multiple attempts to combine angiotensin-converting enzyme inhibitors with angiotensin II receptor antagonists. However, the high risk of kidney injury associated with excessive blockade suggests that this combination should be reserved for situations of high metabolic risk, such as diabetic patients with normal renal function [5]. Similarly, inhibiting other previously discussed molecules, such as interleukins and tumor necrosis factors, which contribute to injury, may inspire further investigation into other potential therapies [7].

Certain secondary forms of arterial hypertension, such as primary aldosteronism, are associated with a significantly higher overall risk of vascular complications [4,8]. Accelerated aldosterone-induced vasculopathy underlies this increased risk of organ failure. Aldosterone modulates sodium kinetics, inducing systemic inflammation and fibrosis, leading to premature atherosclerosis [8]. Primary aldosteronism is closely linked to cardiovascular, cerebrovascular, and metabolic complications, necessitating a more aggressive management approach compared to essential hypertension [8]. Although not clearly defined in European guidelines, we suggest considering a lower threshold for surgical management in this subgroup of patients. Some scoring systems can assess the individual benefit of surgical treatment in certain forms of primary hyperaldosteronism, supporting our opinion [2,4].

Our study has several limitations that must be considered. Our study is retrospective, which means that it relies on past data. This may introduce biases and limitations in data collection and analysis, as it is challenging to control for confounding variables that may influence the results. Our study included a relatively small sample size of 73 patients, which may limit the generalizability of clinical findings. Additionally, the study was performed at a single tertiary hospital care center, which may not fully represent the broader population of hypertensive patients. As we previously mentioned, patients were specifically selected from a specialized consultation for arterial hypertension, which may introduce selection bias and influence the prevalence of end-organ damage. The study data collection period spanned from July 2022 to March 2023, which might not fully capture seasonal variations or long-term trends in hypertension and end-organ damage. We only presented data on the most prescribed drugs, potentially missing important information about medication adherence and patient response to treatment. The study did not account for potential confounding factors (e.g., lifestyle factors, comorbidities, and socioeconomic status), which might influence the observed associations between hypertension and end-organ damage.

## Conclusions

In conclusion, arterial hypertension (AH) remains a significant health issue, emphasizing the need for research to discover new pharmacological classes. Uncontrolled blood pressure increases the risk of target organ damage over time, underscoring the gravity of this condition. Strict blood pressure control, especially in younger patients, is crucial to reduce end-organ damage, which can have profound implications for patients' quality of life and long-term health outcomes. Secondary forms of AH, although less prevalent, require specialized consultations for accurate diagnosis and treatment. Understanding the complex pathophysiology of AH, including immune mechanisms and cytokine release, highlights the importance of reducing inflammation to prevent end-organ failure. The renin-angiotensin-aldosterone system is a promising therapeutic target, and agents blocking this system are effective in blood pressure control and organ protection.

Moreover, primary aldosteronism, a secondary form of AH, warrants a more aggressive management approach due to its association with higher vascular complications, further underlining the significance of tailored interventions. Surgical interventions may be considered earlier in this subgroup to prevent or mitigate potential end-organ failures. Overall, strict blood pressure control and targeted therapies are essential in managing AH and improving patient outcomes. Future research may benefit from expanding the dataset to include additional demographics and exploring links between secondary diseases such as obstructive sleep apnea (OSA) and hypertension while ensuring that the conclusion aligns with the stated study objectives and considers the prevalence of end-organ outcomes as outlined in the objectives.
